# Short- and mid-term efficacy of percutaneous ethanol injection in cystic and predominantly cystic thyroid nodules: systematic review and meta-analysis

**DOI:** 10.1007/s40618-026-02927-y

**Published:** 2026-06-16

**Authors:** Sium Wolde Sellasie, Maurilio Deandrea, Stefano Amendola, Tommaso Piticchio, Luigi Uccioli, Pierpaolo Trimboli

**Affiliations:** 1 Division of Endocrinology and Diabetes, Department of Surgical Sciences, CTO Andrea Alesini Hospital, University of Rome Tor Vergata, Rome, 00133 Italy; 2 School of Applied Medical‑Surgical Sciences, University of Rome Tor Vergata, Rome, 00133 Italy; 3 UO Endocrinologia, Diabetologia e Malattie del metabolismo, AO Ordine Mauriziano, Torino, 101128 Italy; 4 Department of Medicine and Surgery, Kore University of Enna, Enna, Italy; 5 Faculty of Biomedical Sciences, Università della Svizzera Italiana (USI), Lugano, Switzerland; 6 Clinic for Endocrinology and Diabetology, Ente Ospedaliero Cantonale, Bellinzona, Switzerland

**Keywords:** Percutaneous ethanol injection, Thyroid nodules, Cystic nodules, Volume reduction rate, Meta-analysis

## Abstract

**Objective:**

To evaluate the short- and mid-term volume reduction rate(VRR) after percutaneous ethanol injection(PEI), at 1,3 and 6 months, in patients with cystic or predominantly cystic thyroid nodules(CNs/pCNs, respectively), conducting a systematic review and meta-analysis of published data on VRR outcomes across these intervals.

**Design and methods:**

A systematic search of articles published up to October 30,2025 identified studies reporting PEI treatment for CNs/pCNs.Characteristics of the study design, CNs/pCNs cohorts, and outcomes of interest(VRR at 1,3 and 6 months of follow up)were extracted.Statistical analysis included a random-effects meta-analysis, assessment of heterogeneity with use of the I^2^ statistic, and meta-regression and subgroup analyses to explore potential sources of heterogeneity.

**Results:**

Six studies comprising 431 CNs/pCNs were included.The pooled VRRs at 1,3 and 6 months post-PEI were 85.18%(95% CI: 80.72–89.64),91.50%(95% CI: 88.88–94.12) and 93.11%(95% CI: 90.91–95.31),respectively. Stratifying by nodule cystic composition, the VRR at consecutive follow-up time points was significantly different between the 1- and 3-month intervals in both subgroups [CN: VRRs at 1, 3 and 6 months were 91.16% (88.38–93.93), 95.69% (94.16–97.22) and 96.02% (94.16–97.87), respectively; pCN: VRRs at 1,3 and 6 months were respectively 80.19% (77.04–83.33), 87.08% (84.95–89.2) and 90.01%(88.83–91.19)].A secondary meta-regression analysis with baseline mean volume as covariate demonstrated a significant inverse association with VRR at 1 and 3 months in pCN(*p* = 0.02).

**Conclusion:**

By providing pooled VRRs for the short- and mid-term follow-up, this meta-analysis should be regarded as an initial step, paving the way for larger, high-quality studies aimed at standardizing the PEI procedure and supporting its incorporation into future dedicated guidelines.

**Supplementary Information:**

The online version contains supplementary material available at https://doi.org/10.1007/s40618-026-02927-y.

## Introduction

Cystic and predominantly cystic thyroid nodules (CNs and pCNs, respectively) are frequently encountered in the daily clinical practice of thyroidologists [1]. These nodules usually develop from haemorrhage or degenerative changes in previously solid nodules; primary cysts are rare, accounting for approximately 1% of all cases [2]. The risk of malignancy in these lesions is very low (< 1%); therefore, current guidelines [3, 4] do not recommend fine-needle aspiration (FNA) cytology unless treatment is being considered.

Treatment is indicated when CNs/pCNs cause compressive symptoms or aesthetic concerns, depending on their size and location. In such cases, percutaneous fluid aspiration represents the initial non-surgical option and also provides material for cytological evaluation. However, most CNs recur after fluid removal [5]. In the past, surgery was the only available treatment, but given the pre- and postoperative costs and potential complications, it is often considered excessive. In recent years, as suggested by multidisciplinary guidelines [3, 6], minimally invasive techniques have emerged as valid alternatives. Specifically, for CNs/pCNs, percutaneous ethanol injection (PEI) is the recommended option.

PEI is a chemical ablation technique, in which ethanol induces tissue destruction through (I) coagulative necrosis and (II) vascular thrombosis with subsequent ischemia [7]. The therapeutic efficacy of PEI is well established, with volume reduction rates (VRRs) of up to 77% and 81% at 6 and 12 months after treatment, respectively [8].

Owing to its increasing use and proven efficacy, several professional societies have issued PEI-specific guidelines. Notable examples include the recommendations of the Korean Society of Thyroid Radiology [9] and the recent international multidisciplinary consensus statement [6]. These guidelines provide practical indications on patient eligibility, procedural technique, perioperative management, and post-treatment follow-up. For follow-up, the most recent international consensus recommends ultrasound (US) evaluations at early (< 3 months), intermediate (3–6 months), and late (12 months) intervals [6]. While this schedule appears reasonable, it remains largely empirical and has not yet been validated by direct evidence.

In particular, while the long-term efficacy of PEI is well supported by several studies—confirming sustained VRR at late follow-up intervals [8]—evidence regarding short- and mid-term outcomes remains limited. Few studies have reported VRR within the first six months after PEI; therefore, despite the guideline recommendations for short- and mid- US assessments, the effectiveness of PEI at these stages is not well established.

Assessing VRR in the short- and mid-term is essential for defining an evidence-based follow-up schedule. This would not only optimize patient management and institutional resource planning but also standardize inter-institutional comparisons, facilitate collaborative audits endorsed by scientific societies, and reduce unnecessary costs for both patients and healthcare systems.

The primary objective of this study was to evaluate the short- and mid-term VRR after PEI (at 1, 3, and 6 months) in patients with CNs/pCNs. To this purpose, we conducted a systematic review and meta-analysis of published data on VRR outcomes across these intervals.

## Materials and methods

### Review design

The systematic review was conducted in accordance with the PRISMA 2020 statement [10]. The study protocol was registered on PROSPERO (Registration No. CRD420251182148). This study is a systematic review and meta-analysis of previously published data and did not involve any new studies with human participants or animals performed by any of the authors. Therefore, Institutional Review Board approval was not required.

### Search strategy

A comprehensive literature search was performed to identify all original English-language studies reporting PEI treatment for pCN/CNs. The search algorithm used for this research was composed of the combination of these terms: (A) thyroid nodules AND (B) percutaneous ethanol injection. Searches were conducted using PubMed and Google Scholar, with the most recent search completed on October 30, 2025. Additionally, reference lists of relevant studies were manually screened to identify further eligible articles. Two investigators (S.W.S. and P.T.) independently conducted the search, screened titles and abstracts, reviewed full-text articles, and determined eligibility. Discrepancies were resolved through consensus-based discussion.

### Study selection

PEI is an established therapeutic modality for managing symptomatic CN/pCNs. Accordingly, this systematic review included only original research articles reporting comprehensive data on CN/pCNs treated with PEI, with a minimum follow-up period of 6 months. Studies were required to provide US assessments at both 1, 3 and 6 months post-PEI and report VRR or nodule volume (in mL or cm³) as mean ± standard deviation.

The exclusion criteria were as follows: (a) studies outside the scope of the review; (b) studies evaluating PEI in malignant thyroid nodules; (c) studies in which autonomously functioning nodules comprised more than 25% of the CN/pCNs cohort; (d) studies with a dropout rate exceeding 25% at the 6-month follow-up; (e) studies in which PEI had already been performed at baseline; (f) studies with overlapping patient or nodule data; and (g) reviews, editorials, letters, commentaries, or case reports/series. Two independent reviewers (S.W.S. and P.T.) screened the titles and abstracts of all retrieved articles using the above eligibility criteria.

### Data extraction

Primary and supplementary data were extracted and analyzed from the included studies. The following variables were independently collected: general study characteristics (first author, year of publication, country, and study design), number and cystic composition (pCN or CN) of nodules, baseline nodule volume, PEI technique and VRRs at 1, 3 and 6 months post-PEI. In cases where VRR was not directly reported, it was calculated using the usual formula: VRR (%) = ([Initial Volume − Final Volume]/Initial Volume) × 100. When data were expressed as median and IQR, the estimated mean and standard deviation of the sample were calculated using specific formulas [11, 12]. When essential data were missing, the corresponding authors were contacted to obtain the required information. Any discrepancies identified during the data collection process were resolved through discussion among the investigators.

### Study quality assessment

The methodological quality and risk of bias of the included studies were independently assessed by two co-authors using the National Heart, Lung, and Blood Institute Quality Assessment Tool for Observational Cohort and Cross-Sectional Studies.

The tool evaluated key domains, including the clarity of research questions, eligibility criteria, sample size justification, intervention description and implementation, outcome measurement definitions, follow-up duration, blinding procedures, attrition rates, and statistical methodology. Each item was rated as “low risk,” “high risk,” or “not reported” based on the Cochrane Handbook for Systematic Reviews of Interventions.

### Statistical analysis

The initial outcome of interest was the pooled VRR at 1, 3, and 6 months post-PEI, calculated via separate meta-analyses. All pooled estimates were reported with 95% confidence intervals (95% CIs). Heterogeneity among studies was assessed using the I² statistic, with values ≥ 50% indicating substantial heterogeneity. When applicable, significant heterogeneity was further investigated using meta-regression or subgroup analyses.

The primary study endpoint was based on a comparative analysis of VRRs across the different time points. Differences were considered statistically significant when the respective 95% CIs did not overlap. If baseline nodule volumes were not explicitly provided, they were estimated using the reported median values.

Publication bias was evaluated using Egger’s regression test. The potential impact of publication bias was estimated using the trim-and-fill method. A two-sided p value < 0.05 was considered statistically significant. All statistical analyses were performed using Open Meta [Analyst], an open-source software developed by the Center for Evidence Synthesis in Health at Brown University.

## Results

### Study selection

**Fig. 1:**
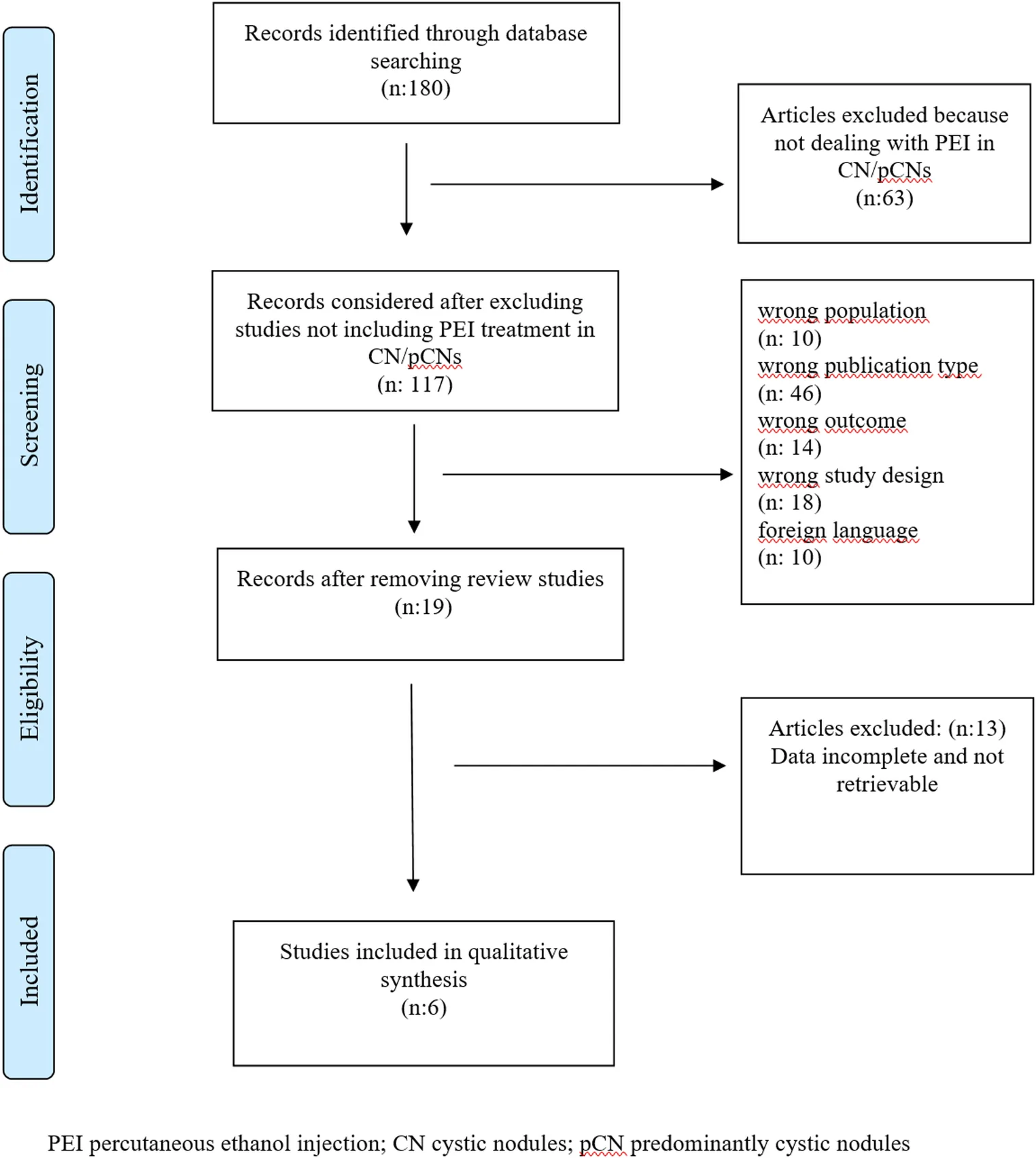
Flow diagram of the study selection process

A total of 180 records were initially identified through the search strategy. After removing duplicates and screening the titles and abstracts, 19 articles were retrieved for full-text review. Of these, 13 studies were excluded due to either unclear or inconsistent data regarding the treated nodules. Ultimately, six studies [13–18] were included in this systematic review (Fig. 1).

### Study quality assessment

The risk of bias in the included studies is detailed in the Supplemental Material Table S1. Overall, six of the 12 assessed domains were rated as having a low risk of bias across all studies. However, no study blinded outcome assessors to the participants’ interventions (item 8), and none employed an interrupted time-series design (item 11). In summary, the half of the included studies were classified as being of good quality, with the others rated as fair quality.

### Qualitative analysis (systematic review)

The six studies included in this systematic review were published between 2015 and 2024. All the studies included were single-center studies. All included studies were observational; one was prospective and single-center, whereas the remainder were based on retrospective analyses conducted at the end of the study period. .

In terms of geographical distribution, 5 studies were conducted in Europe (Czech Republic [*n* = 3] and Spain [*n* = 2]), and the remnant one in China. In total, the study pooled 431 thyroid nodules, 245 pCNs (56.8%) and 186 CNs (43.2%) treated with PEI. The sample sizes ranged from 32 to 200 pCN/CNs per study. The mean baseline volume of nodules, reported in all the 6 studies, ranged from 9.12 to 15.17 mL. The majority of the studies included, the PEI technique did not remove the ethanol previously injected into the cyst. No studies reported major post-PEI complications; only rare, minor and transient adverse events, such as dysphonia or hyperthyroidism, were observed, with recovery occurring within a few days. Mild localized pain was reported in 22% to 30% of cases. Therapeutic success was defined as a VRR ≥ 50% in five studies, with rates ranging from 81.2% to 100%. In one study, therapeutic success was instead defined as complete cyst disappearance and was achieved in all cases. Notably, in the study reporting the lowest therapeutic success rate, all included nodules later achieved therapeutic success after additional PEI sessions. Table 1 summarizes the general characteristics of the included studies.

**Table 1:** General characteristics of the 6 included studies

First author	Year	Country	Journal	Design	Number of nodules	CN, pCN	Ethanol removal	Baseline volume, mL (mean ± SD)	Post-PEI complications	Therapeutic success of PEI
Halenka, M1^13^(a)(b)	2015	Czech Republic	Biomedical papers	RM	1122	CN pCN	Left in the cyst	10.7 ± 510.9 ± 5.2	-no major complications;-mild/morederate localized pain (30%)	100%
Gong, X^14^	2017	China	Journal of Ultrasound in Medicine	RM	70	CN	Removed after10 minutes	15.17 ± 19.1	-no major complications;- mild localized pain (25.9%)	95.2%
Halenka, M2^15^(a)(b)	2020	Czech Republic	Biomedical papers	RM	49151	CN pCN	Left in the cyst	9.12 ± 7.6310.4 ± 7.48	-no major complications;-mild localized pain (29%)- Dysphonia (1%)	100%
Merchante Alfaro, AÁ^16^(a)(b)	2021	Spain	Endocrinología, Diabetes y Nutrición	RM	1617	CN pCN	Left in the cyst	13.06 ± 7.8812.28 ± 4.44	-no major complications;-mild (39%) or moderate (12%) localized pain	100%
Halenka, 3^17^(a)(b)	2023	Czech Republic	European Thyroid Journal	RM	1548	CN pCN	Left in the cyst	13.53 ± 13.5710.31 ± 7.45	-no major complications;-mild localized pain (22%)	100%
Bodoque Cubas, J^18^(a)(b)	2024	Spain	Endocrinología, Diabetes y Nutrición	PM	257	CN pCN	Removed after5-10 minutes	13.27 ± 13.3414.49 ± 22.55	-no major complications;-mild localized pain (% not reported)- transient hyperthyroidism (3.1%)	81.2%

The VRR at 1 month post-PEI ranged from 74.53%, registered in a pCN subseries, to 94.4%, registered in a CN subseries. The VRR at 3 months post-PEI ranged from 83%, registered in a pCN subseries, to 98.3%, registered in a CN subseries. Finally, the VRR at 6 months post-PEI, ranged from 88.4%, registered in a pCN subseries, to 99.3%, registered in a CN subseries (Table 2).

**Table 2:** VRR outcomes at various follow-up points across included studies

First author	VRR, % (mean ± SD)		
	1 month	3 months	6 months
Halenka, M1 ^13^ (a) (b)	89.4 ± 8 79.9 ± 5.2	94.3 ± 4.2 87.7 ± 5.9	87.7 ± 5.9 90.6 ± 6.5
Gong, X ^14^	75 ± 41.07	89.32 ± 16.89	95.72 ± 7.27
Halenka, M2 ^15^ (a) (b)	93.64 ± 5.34 84.15 ± 12.12	96.41 ± 2.59 89.31 ± 10.47	97 ± 2.74 90.59 ± 10.02
Merchante Alfaro, AÁ ^16^ (a) (b)	90 ± 13 74.53 ± 10.5	94.19 ± 8.94 83 ± 8.08	95.46 ± 8.94 88.44 ± 11.31
Halenka, M3 ^17^ (a) (b)	94.4 ± 3 80.2 ± 10.5	96.6 ± 2.5 86.8 ± 9.7	97.2 ± 2 88.4 ± 9.7
Bodoque Cubas, J ^18^ (a) (b)	90.47 ± 15.72 78.79 ± 23.68	98.30 ± 3.16 85.29 ± 12.74	99.33 ± 1.35 88.59 ± 8.30

VRR, volume reduction rate. The alphabetic letters indicate the subseries of the study

### Quantitative analysis (meta-analysis)

The pooled VRRs at 1-, 3-, and 6-months post-PEI were 85.18% (95% CI: 80.72–89.64), 91.50% (95% CI: 88.88–94.12) and 93.11% (95% CI: 90.91–95.31), respectively. The findings from the primary endpoint analysis are presented in Table 3.

**Table 3:** Primary outcome of our meta-analysis

Time after PEI therapy (months)	VRR (%)	95% CI	Significant variation of VRR with respect to the previous one
1	85.18	80.72–89.64	—
3	91.50	88.88–94.12	No
6	93.11	90.91–95.31	No

95% CI, 95% confidence interval; PEI, percutaneous ethanol injection; VRR, volume reduction rate. Data represent the pooled results of the meta-analysis. A change was considered statistically significant when the 95% CIs of successive time points did not overlap

Among all the nodules included, no significant differences in pooled VRR were observed between consecutive follow-up time points. Only between 1 month and 6 months, the VRR differences were significant. Moreover, the 95% CI range narrowed progressively with each time point, demonstrating increased consistency at the 6-month follow-up.

**Fig. 2:**
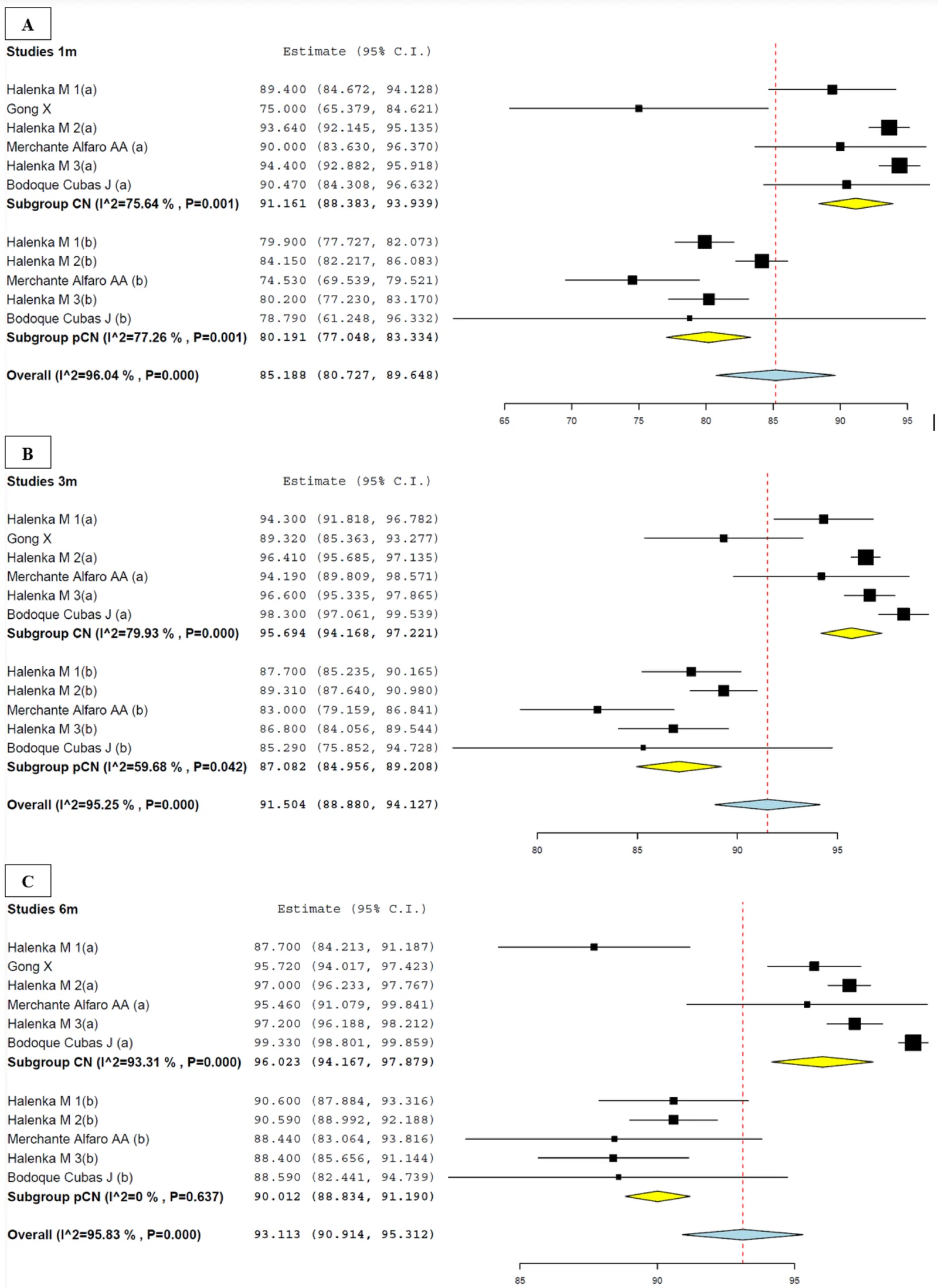
displays the forest plots for the pooled results at 1 month (**A**), 3 months (**B**), and 6 months (**C**) from all six studies, according to the two subgroups (CN vs. pCN)

Considering the heterogeneity, the pooled data were stratified by nodule cystic composition into CN and pCN, applying a meta-regression. The VRR at consecutive follow-up time points was then calculated, revealing a significant difference between the 1- and 3-month intervals in both subgroups, as shown in Table 4. As expected, PEI in CN achieved the highest VRR values across all follow-up intervals (Fig.2).

**Table 4:** Primary outcome of our meta-analysis according to nodules’ cystic composition

Time after PEI therapy (months)	CNs			pCNs		
	VRR (%)	95% CI	Significant variation of VRR with respect to the previous one	VRR (%)	95% CI	Significant variation of VRR with respect to the previous one
1	91.16	88.38–93.93	—	80.19	77.04–83.33	—
3	95.69	94.16–97.22	yes	87.08	84.95–89.2	yes
6	96.02	94.16–97.87	no	90.01	88.83–91.19	no

95% CI, 95% confidence interval; PEI, percutaneous ethanol injection; VRR, volume reduction rate; CN, cystic nodules; pCNs, predominantly cystic nodules. Data represent the pooled results of the meta-analysis. A change was considered statistically significant when the 95% CIs of successive time points did not overlap

Figure 2. Forest plots for meta-analysis of VRR at 1 month (**A**), 3 months (**B**) and 6 months (**C**) following PEI. The size of each square represents the weight of the study, while the diamond indicates the pooled effect estimate with a 95% confidence interval (95% CI). Subseries within studies are marked by letters.

**Fig. 3:**
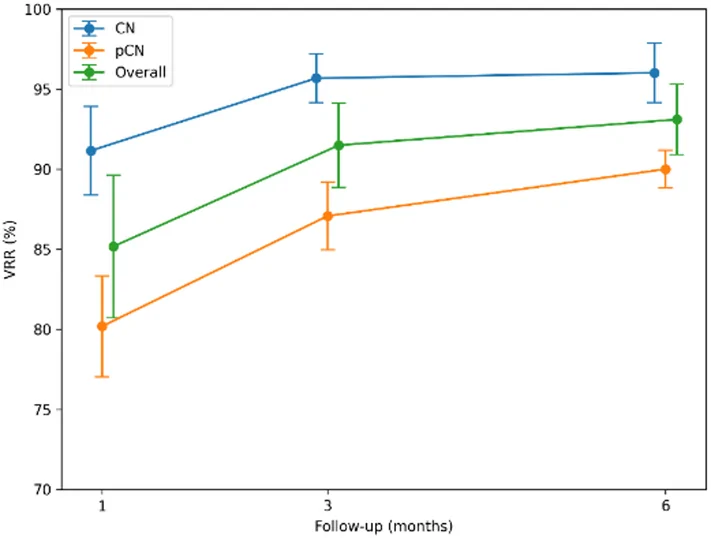
VRR trends across follow up intervals, in the overall cohort and according the two subgroups; CN, cystic nodules; pCN, predominantly cystic nodules

A statistically significant difference in VRR trends between CN and pCN was observed across all follow-up time points, as summarized in Fig. 3.

**Fig. 4:**
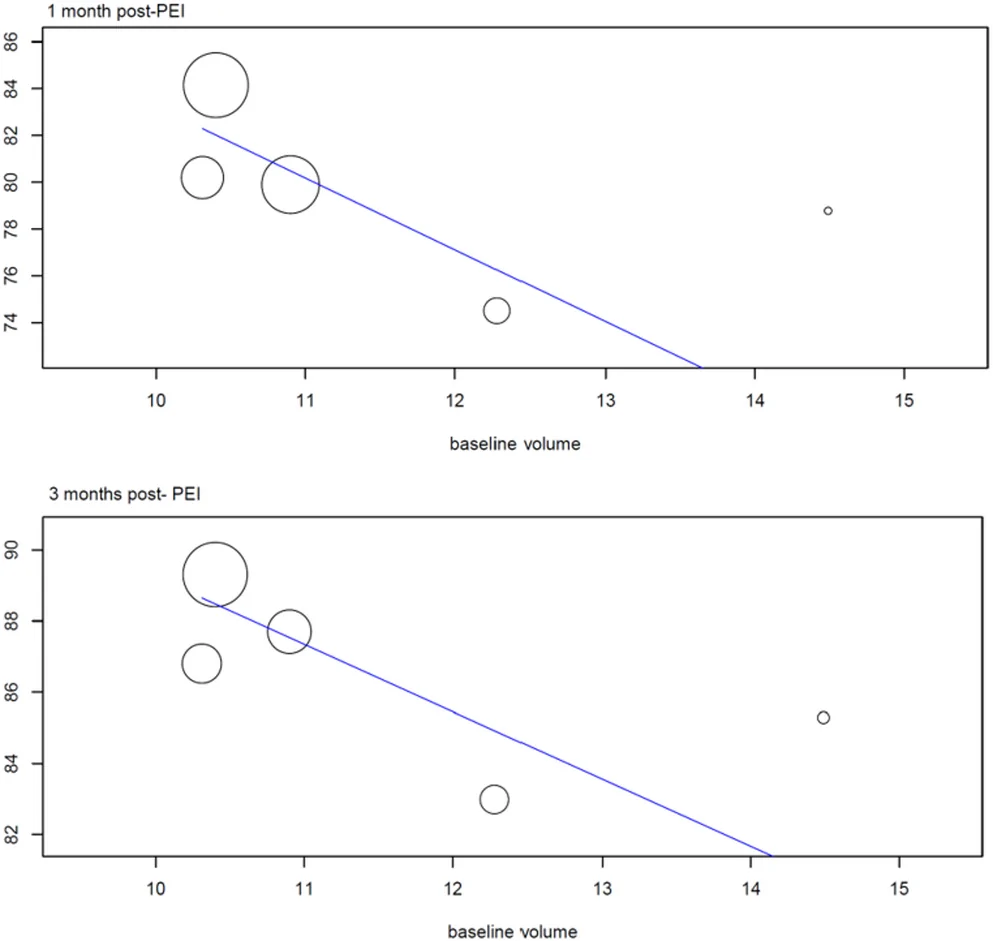
Meta-regression analysis assessing the influence of baseline mean pCN volume on VRR at 1 and 3 months post-PEI. Each circle represents a study, and its size reflects the study’s weight in the analysis

Accounting for nodule cystic composition, a secondary meta-regression analysis with baseline mean volume as covariate demonstrated a significant inverse association with VRR at 1 and 3 months in pCN (*p* = 0.02; Fig. 4). No significant association was detected at 6 months in pCN (*p* = 0.59) or at 1 (*p* = 0.18), 3 (*p* = 0.41), and 6 (*p* = 0.45) months in CN.

## Discussion

PEI is a safe and effective procedure that has gained broad consensus among thyroidologists, becoming the first-line treatment for benign pCN/CN [19]. Given its increasing global adoption, optimizing post-treatment follow-up protocols is of substantial clinical importance. Current international guidelines [6] recommend evaluations at early, intermediate, and late intervals; however, these recommendations are not supported by robust evidence or cost-effectiveness analyses, particularly in the early and intermediate phases. Therefore, it is essential to assess whether follow-up schedules can be streamlined to avoid unnecessary assessments and associated costs, considering also the lower cost of PEI compared with other therapeutic techniques, such as surgery.

Although conducting a formal cost-effectiveness analysis is challenging due to marked variability in procedural costs, healthcare policies, and patient preferences across countries and institutions, characterizing VRR and its temporal trajectory may provide clinically meaningful insights. Such evidence could contribute to refining follow-up protocols, determining the optimal timing for additional PEI sessions [20], and informing the selection of alternative therapeutic approaches—such as thermal ablation or surgery—in the event of previous PEI failure. To address this research question, we performed a systematic review and meta-analysis including six original studies comprising 431 CN/pCN. The results are relevant for clinicians performing ethanol ablation, endocrinologists, and patients alike.

The findings of this meta-analysis demonstrated a consistent and progressive reduction in nodule volume over the 6-month follow-up, with the most pronounced effect occurring within the first three months. Differences in VRR according to cystic composition may account for the heterogeneity of response to PEI: although the temporal trend was similar across follow-up intervals, CNs exhibited greater volume reduction compared with pCNs. In addition, baseline volume emerged as a significant covariate influencing VRR at the early follow-up (1–3 months) in pCNs.

These findings confirm the efficacy of PEI in reducing CN/pCN size during short- and mid-term follow-up [21]. Notably, volume reduction can reach approximately 85% as early as one month and up to 95% after six months. Such performance has allowed to establish ethanol ablation as a first-line treatment for CN/pCNs [22]. The early response to PEI underscores the rationale of this study, which aimed to evaluate VRR at short- and mid-term intervals (1, 3, and 6 months)—time points that are often underreported in studies on PEI procedures.

Although the overall response is consistent, the cystic composition of the nodule plays a crucial role in determining the extent of volume reduction after PEI [23, 24]. As shown by previous evidence [25,26], the effect of ethanol—mediated by coagulative necrosis through several pathways [27] —is less effective on the solid portions of pCN. This consideration is clinically relevant, since the primary goal of ethanol ablation is not only to reduce nodule volume and prevent recurrence, but also to alleviate aesthetic concerns and relieve local compressive symptoms [28]; thus, in some cases, particularly for pCN, the utility of PEI may be limited.

Conversely, baseline volume appears to influence the volume reduction in pCN during the first three months, with smaller nodules showing greater and earlier benefit from PEI treatment [23]. Although a similar trend was observed for CN, as previously reported in a meta-analysis by Cesareo R. et al. [8], which included studies with longer follow-up (> 6 months), this association did not reach statistical significance in our meta-analysis. These differences is likely attributable to several factors, including (I) the lower baseline mean volume in our cohort and (II) the shorter follow-up period considered.

Differences in technical aspects of the PEI procedure remain a major caveat, particularly regarding (I) the volume of ethanol injected and (II) whether or not the injected ethanol is removed from the cyst [29]. The lack of high-quality studies addressing these issues is concerning, and as a result, current guidelines are still unable to provide clear recommendations. As outlined in the qualitative analysis, the studies included in this review exhibit several methodological limitations, notably their retrospective and single-center design, which result in VRR estimates derived from evidence of limited quality. To help address this shortcoming, the pooled data generated through this meta-analysis may offer more reliable estimates of short- and mid-term post-PEI VRRs for thyroidologists and other stakeholders involved in ethanol ablation. In light of these considerations, this systematic review and meta-analysis should not be regarded as an immediate or definitive benchmark for clinical practice; rather, it should be viewed as a preliminary reference point that underscores the need for future investigations—ideally employing more rigorous study designs—to validate and expand upon the present findings.

From a clinical perspective, this work provides a tool to evaluate procedural effectiveness. Institutions are encouraged to implement intra- and inter-institutional audit programs, endorsed by thyroid societies, to monitor outcomes and identify areas for improvement. Moreover, if confirmed by larger future studies, these findings may not only support a simplified follow-up strategy—enhancing resource utilization, reducing unnecessary imaging, and improving patient convenience—but also offer clear guidance on when, how, and where PEI should be repeated to maximize its effectiveness. Alternatively, if PEI proves ineffective—particularly in pCNs—a careful reassessment of the clinical and technical context may justify proceeding with an additional minimally invasive treatment modality, such as thermal ablation [30, 31]. Moreover, future research should instead explore related aspects such as patient-reported outcomes, quality of life, or the development of predictive models for treatment response. Finally, it should be emphasized that, to ensure these levels of performance in terms of VRR and safety, and to maintain the high reliability and quality of future studies, this technique should always be performed in experienced thyroid centers, where PEI can ideally be carried out by qualified thyroidologists, thereby limiting its inappropriate application.

### Limitations and strengths

Several limitations should be acknowledged. First, the observed heterogeneity in the pooled data may reflect unmeasured variables, including operator experience, the amount of ethanol injected, or differences in PEI technique, which is not standardized across studies. As noted above, current guidelines provide no recommendations regarding technique. In fact, only two studies reported ethanol removal from the cysts—one after 10 min [14] and the other after 5–10 min [18]. However, a single retrospective study specifically compared these two approaches and found no significant difference in VRR at one year [29]. Second, only a minority of the included studies had a prospective design; all were single-center, and three enrolled fewer than 50 nodules, underscoring the need for larger, multicenter investigations of PEI efficacy. Third, analyses stratified by baseline nodule volume may be biased, as the mean baseline volume across studies was < 20 ml, which may not represent high-volume nodules. Forth, this metanalysis investigated the different efficacy of PEI in CNs and pCNs. Regarding the latter, the definition is not uniform across studies. In three studies, the cystic component relative to the solid portion was required to be greater than 50% [15, 16, 18]. In another study, this threshold was set at greater than 60% [17], while in one further study it had to be greater than 70% [13].

Despite these limitations, this study is the first to systematically report short- and mid-term (1, 3, and 6 months) VRR after PEI, thereby providing valuable insights for clinicians as well as a reference point for future studies and guideline development.

## Conclusion

This meta-analysis demonstrated a progressive and sustained reduction in nodule volume after PEI for both pCNs and CNs, in the first 6 months post-treatment. The reduction was significantly greater in CNs, although in both subgroups the most pronounced decrease occurred within the first three months. By providing pooled VRRs for the short- and mid-term follow-up, this meta-analysis should be regarded as an initial step, paving the way for larger, high-quality studies aimed at standardizing the PEI procedure and supporting its incorporation into future dedicated guidelines.

## Electronic Supplementary Material

Below is the link to the electronic supplementary material.

Supplementary Material 1

## Data Availability

The datasets generated during and/or analysed during the current study are available from the corresponding author on reasonable request.
