# Supplementary material for: Short- and mid-term efficacy of percutaneous ethanol injection in cystic and predominantly cystic thyroid nodules: systematic review and meta-analysis
Source: J Endocrinol Invest. 2026 Jun 16;49(11):3147–56. doi: 10.1007/s40618-026-02927-y (PMC13722720; doi:10.1007/s40618-026-02927-y)
Supplement: Supplementary file 1 [file 40618_2026_2927_MOESM1_ESM.docx]

**Supplemental materials**

1. Supplemental Table 1: Risk of bias summary: review of authors’ judgements about each risk of bias item for each included in the meta-analysis.

**Supplemental Table 1. Risk of bias summary: review of authors’ judgements about each risk of bias item for each study included in the meta-analysis.**

|  | **1** | **2** | **3** | **4** | **5** | **6** | **7** | **8** | **9** | **10** | **11** | **12** |  |
| --- | --- | --- | --- | --- | --- | --- | --- | --- | --- | --- | --- | --- | --- |
| Halenka et al., 2015 | L | L | H | H | H | L | L | H | L | L | H | N.A. | Fair |
| Gong et al., 2017 | L | L | L | H | L | L | L | H | L | L | H | N.A. | Good |
| Halenka et al., 2020 | L | L | L | H | L | L | L | H | L | L | H | N.A. | Good |
| Merchante Alfaro et al., 2021 | L | L | H | L | H | L | L | H | L | L | H | N.A. | Fair |
| Halenka et al., 2023 | L | L | H | L | H | L | L | H | L | L | H | N.A. | Fair |
| Bodoque Cubas et al., 2024 | L | L | H | L | H | L | L | H | L | L | H | N.A. | Good |

L, low risk; H, high risk; N.R., not reported, N.A., not applicable

Questions:

1. Was the study question or objective clearly stated?

2. Were eligibility/selection criteria for the study population prespecified and clearly described?

3. Were the participants in the study representative of those who would be eligible for the test/service/intervention in the general or clinical population of interest?

4. Were all eligible participants that met the prespecified entry criteria enrolled?

5. Was the sample size sufficiently large to provide confidence in the findings?

6. Was the test/service/intervention clearly described and delivered consistently across the study population?

7. Were the outcome measures prespecified, clearly defined, valid, reliable, and assessed consistently across all study participants?

8. Were the people assessing the outcomes blinded to the participants' exposures/interventions?

9. Was the loss to follow-up after baseline 20% or less? Were those lost to follow-up accounted for in the analysis	10. Did the statistical methods examine changes in outcome measures from before to after the intervention? Were statistical tests done that provided p values for the pre-to-post changes?

11. Were outcome measures of interest taken multiple times before the intervention and multiple times after the intervention (i.e., did they use an interrupted time-series design)?

12. If the intervention was conducted at a group level (e.g., a whole hospital, a community, etc.) did the statistical analysis take into account the use of individual-level data to determine effects at the group level?
